# Radiographic performance depends on the radial glenohumeral mismatch in total shoulder arthroplasty

**DOI:** 10.1186/s12891-020-03219-z

**Published:** 2020-04-03

**Authors:** Anita Hasler, Dominik C. Meyer, Timo Tondelli, Tobias Dietrich, Christian Gerber

**Affiliations:** 1 Department of Orthopaedics, University Hospital Balgrist, Forchstrasse 340, 8008 Zurich, Switzerland; 2 Department of Radiology, Kantonsspital St. Gallen, Rorschacher Strasse 95, 9000 Sankt Gallen, Switzerland

**Keywords:** Anatomical total shoulder arthroplasty, Pegged glenoid component, Glenoid loosening, Radiolucencies, Radial mismatch

## Abstract

**Background:**

Optimal radii of curvature of the articulating surfaces of the prosthetic components are factors associated with the longevity of cemented glenoid components in anatomical total shoulder arthroplasty. It was the purpose of this study, to evaluate the radiographic and clinical performance of an anatomical glenoid component of a total shoulder arthroplasty (TSA) with respect to radial mismatch of the glenoid and humeral component.

**Methods:**

In a retrospective study 75 TSA were analyzed for their clinical and radiographic performance with computed tomography by independent examiners using an established methodology. The study group was divided in two groups, one with mismatch < 4.5 mm (n:52) the others with mismatch ≥4.5 mm (n:23) and analyzed for confounding variables as indication, primary or revision surgery, age, gender, glenoid morphology and implant characteristics.

**Results:**

The mean glenohumeral radial mismatch was 3.4 mm (range 0.5–6.9). At median follow-up of 41 months (range 19–113) radiographic loosening (defined as modified Molé scores ≥6) was present in 7 cases (9.3%). Lucencies around the glenoid pegs (defined as modified Molé score ≥ 1) were present in 34 cases (45%). Radiolucencies were significantly associated with a radial mismatch < 4.5 mm (*p* = 0.000). The pre- to postoperative improvements in Subjective Shoulder Value and absolute Constant Score were significantly better in the group with a mismatch ≥4.5 mm (*p* = 0.018, *p* = 0.014).

**Conclusion:**

A lower conformity of the radii of humerus and glenoid seems to improve the loosening performance in TSA. Perhaps cut-off values regarding the recommended mismatch need to be revalued in the future.

## Background

Glenoid loosening is the most frequent long-term complication and the most common reason for revision in anatomical total shoulder arthroplasty (TSA) [1–6]. Radiolucency at the bone-cement interface is a common finding and predictive of glenoid loosening [6–9].

The incidence of radiolucencies around glenoid pegs ranges from 30 to 93% [10, 11] depending on implant, methodology and duration of follow-up. Asymptomatic radiolucent lines and symptomatic loosening are expected to occur with a yearly increase rate of 7.3 and 1.2% respectively [12]. Currently, curved-back glenoids are considered to experimentally [13, 14] and clinically outperform flat-back glenoids [15]. Pegged glenoids appear to perform better experimentally than keeled glenoids [13], with uncertain superiority in clinical performance [11, 12, 16–22]. The radius of curvature of the articulating surface of the glenoid and the humeral components are of particular interest with respect to the so-called radial mismatch defined as the difference between the radius of curvature of the humeral head and the glenoid components [23]. In 2001, Anglin et al. experimentally documented in a rocking-horse test, that a radial mismatch was associated with less displacement of a glenoid component than if the radii were conforming [13]. The multicenter landmark study by Walch et al. then proved that there is a clear association of radial mismatch with better glenoid performance and clinical results and suggested that a radial mismatch of 6 mm to 10 mm is optimal for prevention of loosening [23]. Further biomechanical and human cadaveric studies supported these findings and demonstrated that a radial mismatch of 4 mm closely emulated the passive glenohumeral motion of the natural joint [24–26]. Biomechanical studies suggested that there was also an upper limit for mismatch, which was considered to be around 10 mm [27, 28]. Despite these studies; recommendations for an ideal mismatch are still unavailable.

Different TSA implants on the market have variable recommended mismatches. These range from 3.5-8 mm (Zimmer-Biomet®, Anatomical Shoulder Prosthesis™) [29], 1.4–24.8 (Tornier® Aequalis Ascend flex) [30], to allowed 2–10 mm mismatch (Medacta®, Medacta shoulder system) [31]. Often a wide range of radial mismatch is in line with the possibility to combine glenoid components with various head sizes and vice versa. It was the goal of this study, to evalue if a certain mismatch correlates with better clinical and radiographic performance of TSA in mid-term follow-up.

## Methods

### Patient selection

The investigational review board responsible for our institution approved this study (KEK-ZH-Nr. 2013–0598). Between 1999 and 2012, 111 TSA were performed by the same surgeon or under direct supervision (C.G). The clinical and radiographic outcomes of the period 1999–2001 were previously published [32]. All patients were operated for a degenerative destruction of the glenohumeral joint with pain and/or functional limitations but an intact rotator cuff. An Anatomical Shoulder Prosthesis™ (Zimmer-Biomet®) with an all-polyethylene glenoid component with convex, roughened back surface and four threaded pegs was used in all shoulders. Patients were then excluded if one of the following exclusion criteria was met: Posttraumatic osteoarthritis with tuberosity malunion and/or bony deformity of the glenoid, rotator cuff insufficiency, pre- or postoperative infection, preoperative neurological lesion and shoulder revised before last follow-up.

On the basis of the experimental data of Anglin et al. [13], the radius of curvature of the bearing surface of the polyethylene had been increased to the currently marketed dimensions in 2002. *(*Table 1*)* Neither the dimensions of the glenoid components, nor the form and structure of the backside, nor the shape or structure of the pegs, nor the material of the component were altered. The humeral heads remained identical resulting in a bigger radial mismatch with the implants after 2002. The mean mismatch before 2002 was 2.1 mm respectively 4.7 mm after 2002. This was the reason to build two groups (mismatch < 4.5 mm vs. mismatch ≥4.5 mm) for the analysis of clinical and radiographically outcome as well as the analysis of cofounding variables.

**Table 1:** Radius Glenoid

	Before 2002	After 2002
Glenoid size S	27 mm	29.5 mm
Glenoid size M	29 mm	31 mm
Glenoid size L	29.5 mm	33 mm

**Fig. 1:**
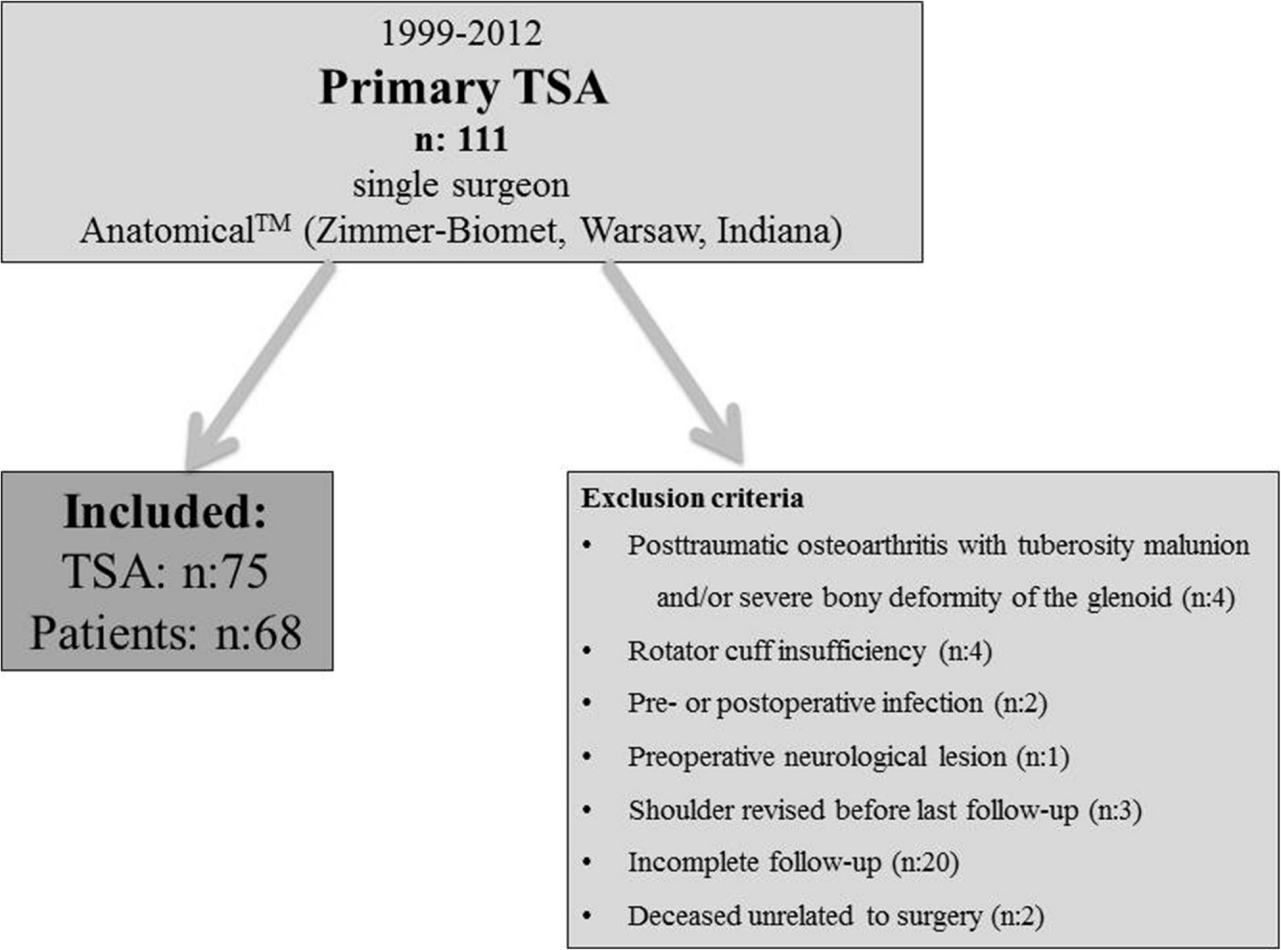
Summary of exclusion criteria and included patients. For the shoulders revised before last follow-up, there was no glenoid loosening visible. Two of these revisions were conversions to reverse total shoulder arthroplasties because of secondary rotator cuff tearing. One further case was revised for humeral loosening; the glenoid was stable and left in place

These changes led to a wide range of radial mismatches in our study population. Because of the narrow criteria, only seventy-five shoulders remained for this retrospective study. *(*Fig. 1*).*

The median follow-up period was 41 months (range 19–113). The median age at index surgery of the total 68 patients (75 shoulders) was 61.7 years (range 26.0 to 84.1) at the time of index surgery (38 women, 30 men). 26 left and 49 right shoulders were included. In 43 cases (57.3%), primary osteoarthritis was the diagnosis. Avascular necrosis and crystalline arthritis were the diagnosis in 12 cases (16%) each, in the remaining 8 cases (10.6%) secondary osteoarthritis was the diagnosis. In 21 cases, this was the primary surgery, in 54 cases the index surgery was at least the second procedure. The mean mismatch was 3.4 mm (range 0.5–6.9). Further implant characteristics can be found in Table 2.

**Table 2:** Implant characteristics

Total (n)	75
**Implant version** (before 2002/after 2002)	45/30
**Glenoid size**	
**S**	20
**M**	43
**L**	12
**Head size (mm)**	
**40**	5
**42**	4
**44**	10
**46**	22
**48**	8
**50**	15
**52**	11
**Mismatch (mm)**^a^	3.4 (0.5–6.9)

^a^ Values in median, with range ()

### Operative Technique

TSA was performed in a standardized fashion [15]. Specifics of glenoid preparation included: First, the whole labrum including the biceps anchor was resected. The cartilage was removed with a sharp curette. Minimal reaming was then performed using a cannulated reamer. Reaming was stopped before the sclerotic bone was removed so that a layer of hard, subchondral bone remained. If an excessive glenoid retroversion (> 10°) was present, the surgeon attempted to correct this by asymmetric reaming of a maximum of ten degrees. In 26 of 33 cases (79%) this aim was achieved, as documented by analysis of pre-and postoperative CT scans. The cement fixation was performed according to Nyffeler [28] with low viscosity bone cement with antibiotics (Allofix-G® (1999–2001) or Palacos-LV + G®. (2002–2012). This change was necessary, because Allofix was not produced anymore.

### Clinical evaluation

Preoperative evaluation was standardized and included scoring according to Constant and Murley (abs. CS) [33]. To consider the age of the patients, the relative, age-adapted Constant scores were used (rel. CS) [34]. By asking the patient to express the value of his operated shoulder as a percentage of a completely normal shoulder, the Subjective Shoulder Value (SSV) was determined [35–38]. The pain level was evaluated as part of the abs. CS from 0 to 15 with 15 point indicating complete freedom from pain. This clinical evaluation was repeated during the study at last follow-up.

### Radiographic evaluation

**Fig. 2:**
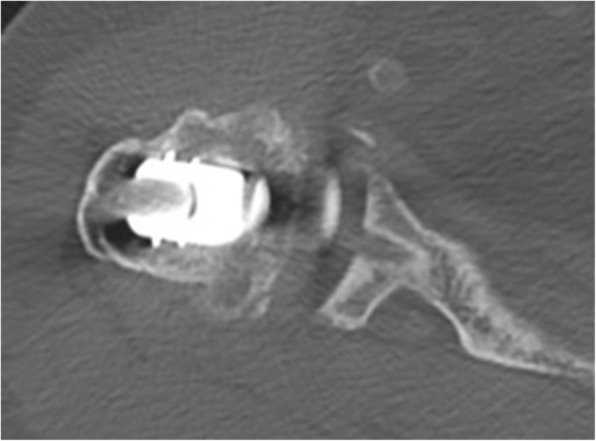
This CT-scan shows a pegged glenoid without any radiolucencies as an example of the image quality. The middle peg (zone 4) is illustrated

**Fig. 3:**
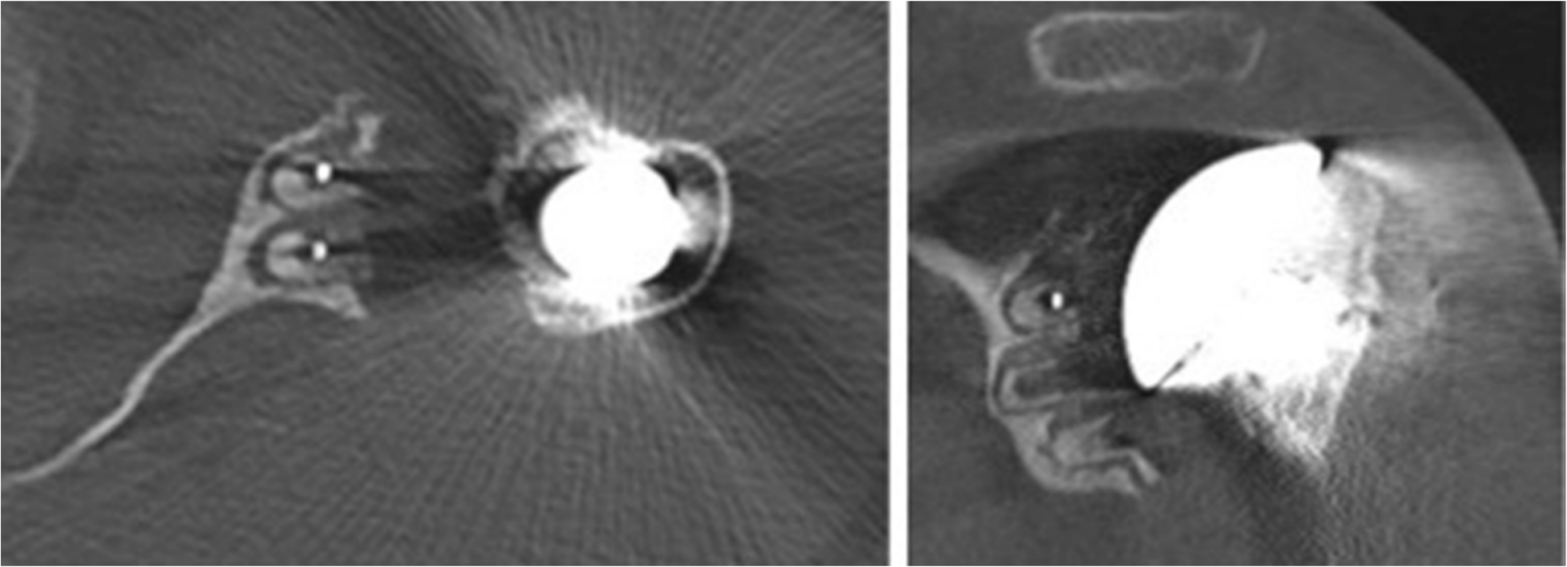
A TSA with a radiolucency score of 18. (≥ 2 mm- wide radiolucency around all pegs and in the zones between)

Native CT- scans were acquired preoperatively (n: 72) and at the most recent follow-up visit for all 75 shoulders. All CT- scans for the follow-up study were performed on a brilliance 64-channel CT-scanner with 2–3 mm contiguous sections from the acromioclavicular joint to the distal third of the scapula (SOMATOM Definition AS). Two separate reviewers interpreted each CT-scan. The examinations were randomly ordered, and the reviewers were blinded to the identities of the patient and the clinical results. According to Friedman and Walch, preoperatively glenoid version and humeral head subluxation [39, 40] were measured. To classify the existence and dimension of radiolucencies, we used the technique of Yian et al. [32]. The glenoid back surface was divided into six zones and it is a numeric assessment of the importance of the radiolucency and includes thickness *(1 mm = 1 point, 2 mm = 2 points, > 2 mm = 3 points)* and location. With this scoring system 0 points were considered no lucencies *(*Fig. 2*)*, equal or more than 1 point was lucencies. Loosening was defined following Yian as modified Molé scores ≥6. (Fig. 3).

### Statistical analysis

Intraclass correlation (two-way random effects) was used to determine interobserver reliability of lucency scores. Box plots of lucency scores appeared non-normal; hence, non-parametric tests were applied. Ordinal and categorical variables were tested by the Mann-Whitney U test and Fisher’s exact test, respectively. The significance level was set at 0.05 and the results are reported as median and range if not stated otherwise. Statistical analyses were computed using Stata/IC 15.1 (StataCorp LP, College Station, TX, USA).

## Results

### Clinical outcome

Preoperative evaluation was incomplete in 6 cases. For all other patients complete data sets were available. In the entire series all clinical outcome parameters improved significantly (all *p* = 0.000). Median abs. CS improved from 40 points (range, 8 to 86) preoperatively to 75 (range, 27 to 98) postoperatively. The median rel. CS increased from 50% (range, 9 to 100) preoperatively to 93% (range, 30 to 110) postoperatively. The median SSV changed from preoperatively 30% (range, 0–85) to 90% (range, 20–100) postoperatively. The median pain level improved significantly from 5 points (range, 0 to 11) to 14 points (range, 0 to 15; 15 points indicating complete freedom from pain).

### Complications

In total, 5 complications were identified. Two of them occurred intraoperatively. In one case a greater tuberosity fracture occurred which was fixed with suture cerclages. Postoperatively an abduction splint was installed for 6 weeks and the fractured healed uneventfully. The other complication was an intraoperative partial nerve lesion of the axillary nerve, which was sutured immediately (dorsal part). At latest follow-up, no functional deficit of the deltoid muscle could be seen. Two patients needed a revision, one subscapularis refixation after a fall and one shoulder arthroscopy with capsulotomy because of capsulitis adhesive. Both patients had no limitations at last follow-up. A seventy-two year old patient sustained a bilateral pulmonary embolism diagnosed one month after surgery. An oral anticoagulation was started and the further course was uneventful.

### Radiographic evaluation

Median glenoid version was − 10° (range, − 35° to 12.4°) and median humeral subluxation was 0.5 (range 0.41–0.74) preoperatively respectively − 6° (range − 24°-14°) and 0.5 (range 0.29–0.59) postoperatively. Postoperatively computed tomography showed radiolucencies in 34 of 75 cases (45%) with a median lucency score of 3.2 points (range, 1 to 18). The most affected zone was zone 5 (inferior peg, ventral). The other zones were affected in the following descending order: zone 2 and 6, zone 4 > zone 1 > zone 3). In seven patients (9.3%) a score of ≥6 identifying loosening of the glenoid were documented.

### Clinical and radiographic performance regarding mismatch

Fifty-two patients had a mismatch < 4.5 mm, respectively twenty-three had a mismatch ≥4.5 mm. To control for potential cofounding variables, we compared the demographic data of these two groups (mismatch < 4.5 mm vs. mismatch ≥4.5 mm). There was no significant difference in age, sex, involved side and primary or revision surgery between the two groups. The duration of the follow-up was significantly longer in the group with mismatch ≥4.5 (*p* = 0.040). The implant characteristics were significantly different in glenoid size (*p* = 0.000). This is due to the fact that with the knowledge, that greater mismatch is associated with less loosening, in case of doubt, the larger glenoid component was selected after 2002, whereas before that often the smaller component had been used. As described above, the size of the humeral head was not changed and therefore, no significant difference could be seen between the two groups (*p* = 0.330). Because change of the cement overlapped with the introduction of the new prosthetic design, this variable was significantly different between the two groups (*p* = 0.000) (Details Table 3).

**Table 3:** Demographic data between mismatch groups

	Mismatch < 4.5 mm	Mismatch ≥4.5 mm	*p*-value ^§^
**Total (n)**	**52**	**23**	
Age at surgery (years)*	61.0 (26–84)	62.5 (41–78)	0.062
**Sex (m/f)**	m:22, f: 30	m:12, f: 11	0.294
**Involved side (l/r)**	l:20, r:32	l:6, r: 17	0.221
**Revision surgery (n)** *(details see below)*	15	6	0.520
Follow-up period (months)*	40 (26–110)	56 (19–113)	**0.040**
**Implant characteristics**			
**Implant version**	before 2002:43 after 2002:9	before 2002:2 after 2002:21	
**Glenoid size**			
**S**	20	0	**0.000**
**M**	30	13	
**L**	2	10	
**Head size (mm)**			
**40**	5	0	0.330
**42**	4	0	
**44**	7	3	
**46**	11	11	
**48**	8		
**50**	11	4	
**52**	6	5	
Mismatch (mm)*	2.6 (0.5–4.2)	4.9 (4.5–6.9)	**0.000**
**Cement**	Allofix-G: 43 Palacos-LV + G): 9	Allofix-G: 1 Palacos-LV + G): 22	**0.000**
* Values in median, with range () or exact values if not applicable *§ Exact fisher or Ranksum test*			
***Details revision surgery:*** *Mismatch < 4.5 mm* *- ORIF proximal humerus fracture (5)* *- Shoulder stabilization surgery (7)* *- Resection heterotopic ossifications (1)* *- Rotator cuff reconstruction (1)* *- Shoulder arthroscopy with biceps tenotomy (1)*		*Mismatch > 4.5 mm* *- Shoulder arthroscopy with acromioplasty (2)* *- ORIF proximal humerus fracture (2)* *- Shoulder stabilization surgery (2)*	

**Fig. 4:**
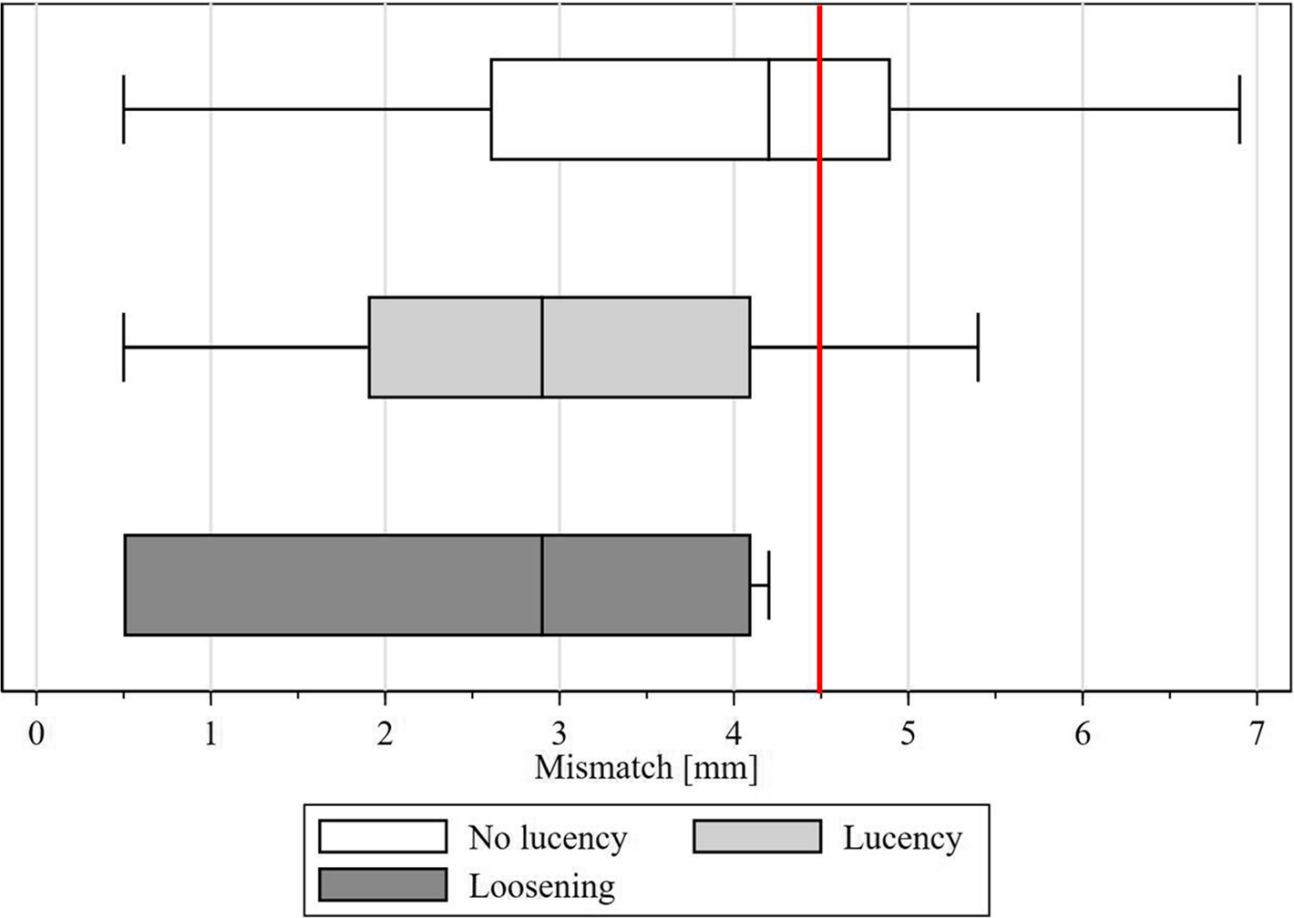
Red line marks the mismatch of 4.5 mm. No loosening occurred with a mismatch ≥4.5 mm

Radiographic preoperative values as glenoid version and preoperative humeral subluxation did not differ between the two groups. Postoperatively, only humeral subluxation was significantly increased in the group with mismatch ≥4.5 mm (p = 0.000). More details are described in Table 3. (Table 4). None of the preoperative and postoperative clinical parameters differed between the two groups but there was a significantly greater improvement in SVV and absolute CS in the mismatch group ≥4.5 mm. (*p* = 0.018 resp. 0.013) *(Details see* Table 5*)*. The group with mismatch < 4.5 mm had significantly (*p* = 0.006) more lucencies (Median 1.3 points, range 0 to 18) at a significantly shorter follow-up than the group with a mismatch of greater than 4.5 mm which had a median of 0 points with a range of 0 to 3.5, but with the available data not significantly more loosenings (*p* = 0.067). As Fig. 4 shows, with a mismatch of at least 4.5 mm, there was no glenoid loosening in our series.

**Table 4:** Radiographic findings in correlation with mismatch

	Mismatch < 4.5 mm	Mismatch ≥ 4.5 mm	*p*-value^§^
**Preoperative glenoid version**^a^	−8.5° (−35°-8°)	−10.3° (−29.4°-12.4°)	0.217
**Postoperative glenoid version**^a^	−6° (−24°-14°)	−5.7° (−16.2°-10.7°)	0.963
**Preoperative humeral subluxation**^a^	0.5 (0.41–0.74)	0.50 (0.44–0.66)	0.177
**Postoperative humeral subluxation**^a^	0.49 (0.29–0.57)	0.53 (0.43–0.59)	**0.000**

^a^ Values in median with () range

^§^Fisher’s eact test or Ranksum test

**Table 5:** Clinical outcome of TSA regarding the mismatch groups

	Mismatch < 4.5 mm			Mismatch ≥ 4.5 mm			*p*-value ^§^		
	Preoperative	Last follow-up	Difference	Preoperative	Last follow-up	Difference	Preoperative	Last follow-up	Difference
**SVV** **(%)**	30 (0–85)	85 (20–100)	40 (−10–85)	30 (0–50)	90 (60–100)	60 (20–90)	0.947	0.121	**0.018**
**Absolute CS** **(points)**	43 (8–86)	74 (27–95)	30 (5–63)	38 (11–75)	77 (46–98)	46 (0–80)	0.325	0.213	**0.013**
**Relative CS** **(%)**	53 (9–100)	94 (30–108)	34.7 (3–77)	48 (12–90)	92.6 (59–110)	48.1 (3–77)	0.514	0.851	0.129
**Pain level** **(points)**	5 (0–11)	14 (0–15)	7.5 (0–15)	5 (1–10)	13 (9–15)	7 (2–14)	0.937	0.741	0.990

All Values in median, with range ()

^§^ exact fisher or Ranksum test

### Correlation clinical outcome and lucency/loosening

The clinical outcome with respect to lucency or loosening is statistically different with a lower absolute CS if radiolucency or radiographic loosening occur (*p* = 0.05). All other clinical finding were not statistically different in loose and stable glenoid components.

### Interobserver reliability

The reliabilities of the computed tomography scoring system were high with an interclass correlation coefficient with 0.93 (design before 2002) and 0.98 (design after 2002) respectively.

## Discussion

Glenoid loosening is the most important factor for long-term failure of TSA [10–12]. Factors for glenoid loosening include integrity of the cuff, type of glenoid wear (concentric, eccentric), technique of cementing, type of implant (pegged or keeled), metal back vs. cemented, and design features such as the glenohumeral mismatch [20]. With a mismatch of > 0, there is only a point contact between the glenoid and the humeral head. In reality, the humeral head will certainly deform the much softer material of the glenoid under load. This creates a small contact area in the form of a spherical surface. Thus, the bigger the mismatch, the smaller the possible contact area or the later it is reached under load. This fact leads to more durability of the bone-cement interface and hypothetically to less radioluceny [27]. The glenohumeral mismatch has been found to be relevant in a large multicenter study on conventional radiographs with a keeled glenoid, however several possibly cofounding factors have not been analyzed there [27]. Due to the multifactorial nature, isolating a single factor is difficult. In the here presented study glenohumeral mismatch on a pegged glenoid was the critical factor for increased radiolucency. Due to strict inclusion criteria and with presumably more sensitive evaluation methods using computed tomography, which also allowed to asses factors such as glenoid version and shoulder centralization and a better evaluation of glenoidloosenig/−lucency [32], this study is to our knowledge unique in the current literature.

Overall we could find radiolucency in 34 of 75 cases (45%) and radiographic loosening in seven shoulders (9.3%). Smaller glenohumeral mismatch correlated with poor radiographic performance and less clinical improvement in SVV and absolute CS. With a mismatch greater than 4.5 mm no radiographic loosening occurred in our cohort. If we compare the clinical outcome regarding the occurrence of any lucency, we could just see a statistical difference between the absolute CS postoperatively, which is lower when radiolucency occurs.

The strengths of our study include that an implant was used for which only the radius of curvature of the bearing was changed and for which the same humeral articulating components had been used. The radiographic measurements were made on computer tomography scans (CT-scans) by two independent observers, blinded to the clinical outcome. The strict application of inclusion and exclusion criteria resulted in a relatively small number of patients, which is a limitation of our study.

Because in the literature, there is no cut-off value for radial mismatch, we formed the group (mismatch < 4.5 mm vs. mismatch ≥4.5 mm) to distinguish between the two design versions of the prothesis. That allowed us to compare the two groups in terms of clinical and radiographically outcome, but of course this does not lead to the establishment of a critical value for radial mismatch.

There is no evidence that surgical technique as well as cementing technique has changed over time, but unconscious changes may have played a role, which makes it difficult to attribute all the differences in the results exclusively to the implant change. In fact in our hospital we had to change the cement in 2002 from Allofix® to Palacos® because first was not produced anymore. It is recognized that the patients with mismatch equal or over 4.5 mm had a mean follow-up which is substantially and significantly longer. With the unquestioned progressive nature of glenoid loosening [12] this strengthens and validates the interpretation of the improvement of our results using a mismatch of more than 4.5 mm. All other demographic data as age, sex, involved side, diagnosis and primary or revision surgery weren’t significant different in the two groups.

## Conclusion

We conclude that a lower conformity of the radii of humerus and glenoid seems to improve the loosening performance in TSA. Perhaps critical values regarding the recommended mismatch need to be revalued and adjusted.

## Data Availability

The dataset analyzed during the current study is not publicly available due to patient protection act but are available in anonymized form from the corresponding author on reasonable request.

## Abbreviations

Abs. CS: Absolute Constant score
CT: Computer Tomography
Rel. CS: Aged-related Constant score
SVV: Subjective Shoulder Value
TSA: Total shoulder arthroplasty
